# Literary Notes

**Published:** 1902-09

**Authors:** 


### LITERARY NOTES.

THE *Medical Standard*, published by G. P. Engelhard & Co., Chicago, will present itself for September as an educational and association number. The subjects will be treated in three main divisions, namely—medical colleges, medical literature and medical societies. The contributors to this number will include some of the most distinguished writers in American medicine, and numerous illustrations will attractively supplement the text. Orders should be placed in advance.

---

The J. B. Lippincott Company, of Philadelphia, having recently completed their building, situated at Sixth and Locust Streets, have equipped it with new and improved machinery, and have exceptional facilities for the typesetting, illustrating, printing, binding, and mailing of weekly and monthly periodicals of every description.

They make a specialty of reports of medical colleges, training schools, hospitals, and asylums, and of the publication of genealogical, historical, medical, and surgical works.

They also make a specialty of the printing of fine editions of poems, translations, essays, and memoirs for private distribution, and of bindings of all varieties from the plainest cloth to the finest levant morocco.

With a thoroughly competent proof-reading department, together with well-qualified operatives in every branch of the business, they can be of great assistance to publishers of technical, trade, or professional works.

Correspondence concerning the manufacture of everything pertaining to book or fine pamphlet work is solicited and will be given prompt attention.

